# Network connectivity between benevolent childhood experiences and uncertainty stress among Chinese university students

**DOI:** 10.3389/fpsyt.2022.1007369

**Published:** 2022-11-01

**Authors:** Yifei Pei, Jingjing Wang, Jie Tang, Na Yan, Yunjiao Luo, Yaofei Xie, Qin Zhou, Caiyi Zhang, Wei Wang

**Affiliations:** ^1^Department of Community and Health Education, School of Public Health, Xuzhou Medical University, Xuzhou, China; ^2^Department of Psychiatry, The Affiliated Xuzhou Oriental Hospital of Xuzhou Medical University, Xuzhou, China; ^3^Key Laboratory of Human Genetics and Environmental Medicine, Xuzhou Medical University, Xuzhou, China; ^4^Center for Medical Statistics and Data Analysis, Xuzhou Medical University, Xuzhou, China

**Keywords:** benevolent childhood experiences (BCEs), uncertainty stress, network analysis, university students, mental health

## Abstract

**Background:**

The purpose of this study was to explore the association between benevolent childhood experiences (BCEs) and uncertainty stress among Chinese university students by network analysis.

**Methods:**

A total of 1,830 university students from three Chinese cities were recruited. Respondents' BCEs and uncertainty stress were self-reported using online questionnaire. The structure of the BCEs-uncertainty stress and related centrality indicators were examined for this sample.

**Results:**

The overall network model showed that “no ways to suit the important changes in life” was the most influential, followed by “all things are not going well,” “feel that there is nothing to do,” and “worry about the future.” And in this network, the most influential bridge symptom was “having a positive self-concept.”

**Conclusion:**

The central symptoms of the BCEs-uncertainty stress network should be prioritized as targets in interventions and prevention efforts to reduce uncertainty stress among Chinese university students. Improving university students' positive self-concept is important to alleviate the level of uncertainty stress among Chinese university students.

## Introduction

University students are passing through a critical transitory period (1, 2). In general, they have to adapt to various social demands besides coping with the academic in preparing for their professional careers (3, 4). Because of psychologically and physically immature, university students might suffer various psychological issues during this period (5–7). Stress, as the representative of the mental health problems of university students, impacts an individual's ability to perform life activities (8). Elevated stress has been reported in more than 75% of college students (9). Moreover, the existing studies revealed that students' performance in university are affected by stress (10–12) and these students have much greater rates of stress than those found in the general population in a systematic review (13). In recent years, uncertainty stress has received more attention from academics, owing to the fact that it can have more serious consequences and effects than life stress and study stress (14, 15). Uncertainty stress is a sort of mental stress induced by uncertainty regarding someone or something (16). It is generally said that the greater the degree of uncertainty in one's life, the less comfortable one is and the more likely one is to experience stress (17). Number of studies have demonstrated that uncertainty stress is a major social and public health problem in China (14, 17). According to a nationwide survey of university students in China, nearly one-third of the students had high levels of uncertainty stress (18). What's more, uncertainty stress influences individual development by testing one's ability to predict and plan (19). As a result, it has become a priority to implementing uncertainty stress reduction programs among university students from the public health perspectives.

Several psychosocial stressors (14) for uncertainty stress among university students have been identified, including father's and mother's occupations, family income, family location, and GDP of the original region, etc. Besides these, negative factors related to a fear of failure, concerns for the future and experiencing stressful events also carry a greater risk of uncertainty stress (20). Recently, researchers have paid more attention to benevolent childhood experiences (BCEs) and see it as one of the psychosocial protective factors. A host of evidence demonstrated that BCEs can help slow the progression of psychiatric illness (such as stress and depression) (21–23). BCEs are positive experiences in which one remembers feeling comfortable, safe, and connected with others (24). Emerging evidence suggests that those who report more BCEs in childhood have better mental health (25). Furthermore, one cross-sectional study of Chinese university students shows that BCEs are negatively associated with uncertainty stress and provide a buffer for uncertainty stress (26). Hou et al. also found that Chinese university students who experienced more BCEs were less likely to have uncertainty stress (27).

Generally, the studies of uncertainty stress always take the uncertainty stress measure as a whole and determine the state of uncertainty stress by calculating composite scores. The importance of measuring not only whether symptoms have changed, but also the interactions between individual symptoms has recently been highlighted by a newly developed symptom network perspective (28–30). So far, unfortunately, no studies have investigated how BCEs and uncertainty stress are related to each other in university students using the network model. Using network analysis to examine the dynamic changes between BCEs and uncertainty stress among Chinese university students could provide a more in-depth understanding. Therefore, the purpose of this study is to use network analysis to explore the relationships between BCEs and uncertainty stress in Chinese university students.

## Methods

### Participants and procedures

Twenty-five universities in three Chinese cities (Xuzhou, Nanjing, and Wuhan) were used to recruit participants. To begin, schools were chosen using a stratified sampling method based on school levels as indicators. A total of 25 universities were selected. Second, classes in each university were selected using a stratified random sampling method based on majors, and then cluster sampling was used in each class. Data were collected by using an online survey platform (www.wjx.com). All participants gave their consent to participate in the study and allowed their data to be used for the research. Finally, from March to May 2021, the online questionnaire interviewed a total of 2022 students. Following the exclusion of individuals with missing data for key variables, an analysis sample of 1,830 was used (response rate: 90.50%). The Medical Ethics Committee of Xuzhou Medical University granted ethical approval for this study.

### Measurements

#### Demographic characteristics

The following data was collected: age (in years), residence status (urban/rural), gender (male/female), grade (freshman/sophomore/junior/senior), monthly disposable funds (in RMB: ≤1000/1001-2000/2001-3000/>3000), and only child (yes/no).

#### Uncertainty stress

The standard Perceived Stress Questionnaire, which has acceptable reliability and validity was used to assess uncertainty stress (16). It has previously been used successfully in studies involving Chinese university students (16, 31). The scale consists of 10 items that assess current life uncertainty, uncertainty about social change, goal uncertainty, and social values uncertainty. The specific items are as follows (1) life is subtle, and fate is unpredictable, (2) all things are not going well, (3) experiencing chaos and confusion, (4) unexpected things often occur in life, (5) the world is changing too quickly, and cannot keep up, (6) do not know how to achieve goals, (7) worry about the future, (8) many people do things without rules and do not know what to do, (9) no ways to suit the important changes in life, and (10) feel that there is nothing to do. All items were rated on a 5-point Likert scale ranging from 1 (no stress) to 5 (excessive stress). The responses were added up to get a total score, with the total score increasing the uncertainty stress. (Cronbach's Alpha = 0.95).

#### Benevolent childhood experiences (BCEs)

The BCEs scale (27), which comprises 10 items of positive childhood experiences that occurred before the age of 18, was used to assess BCEs. Items include (1) having at least one safe caregiver, (2) having at least one good friend, (3) having beliefs that gave comfort, (4) enjoying school, (5) having at least one teacher who cared, (6) having good neighbors, (7) having an adult who could provide support or advice, (8) having opportunities to have a good time, (9) having a positive self-concept, and (10) having a predictable home routine. Each “Yes” answer was recorded as 2 and a “No” answer was recorded as 1. Ten items were added to a total score of BCEs (range = 10–20), and the higher score indicates more positive childhood experiences. The Cronbach's α of the scale was 0.71 in the present study.

### Data analysis

The demographic characteristics of the sample were presented using descriptive statistics by SPSS version 25.0. Next, we performed a network analysis on network estimation and network stability.

#### Network estimation

R program (version 4.1.0) was used to conduct network analysis. Each item is represented as a node in the network analysis layout, and the connection between two nodes is depicted as an edge (29). Correlations are stronger when the edges are thicker. Positive and negative correlations were denoted by blue and red color edges, respectively (28). Pair-wise Pearson correlations were used to estimate the symptom network indicating the association between BCEs and uncertainty stress (32). Using the Enhanced Least Absolute Shrinkage and Selection Operator (eLASSO) approach, the network structure of BCEs and uncertainty stress data was evaluated (33). The R-package “qgraph” (Version 1.6.5) and “bootnet” (Version 1.4.3) were used to estimate and visualize the network (34). The Extended Bayes Information Criterion (EBIC) (i.e., a measure of goodness of fit) was used to select the optimal set of neighbor factors for each node (symptom), and the penalty parameter was utilized to obtain sparsity. The final network is formed automatically and reflects the strength of direct correlations between nodes when each node (a node represents a symptom) is connected to a number of other nodes through edges (an edge represents particular relationships between two symptoms) with various weights (33). Symptoms that had stronger and more frequent connections with other nodes were clustered together in the network. The network structure was defined using network centrality indices, which indicate where each node is located within a weighted network, i.e., strength, closeness, and betweenness. Measures of centrality are presented as standardized values (z-scores). Nevertheless, previous study (35) summarized that Strength was the only centrality index employed in this study since Betweenness and Closeness centrality were inappropriate for psychological networks. Strength is the sum of the weight of all direct associations between a certain symptom and others.

#### Estimation of network accuracy and stability

The R package “bootnet” (Version 1.4.3) was used to conduct all network stability evaluations (36). Firstly, bootstrap tests based on 95% CIs were used to analyze the differences in strength between two edges or two nodes. If zero was not included in the CIs, there were statistical differences between the two edges or nodes (36). Then, based on the 95% confidence intervals (95% CIs), a non-parametric bootstrap approach was employed to evaluate the edge weights stability. Edge accuracy was measured using 95% confidence intervals (CIs), with a narrower CI indicating a more reliable network and a larger CI suggesting inferior accuracy (36, 37). Additionally, to verify network stability, a case-dropping bootstrap procedure was used. During this procedure, an increasing proportion of instances were removed from the dataset, while the centrality indices were re-estimated. A network is stable if a considerable amount of the sample can be removed from the dataset without causing significant changes in the indices, and the stability is measured using the Correlation Stability Coefficient (CS-C) (36). The value of CS-C referred to the maximum proportion of dropped cases to maintain a correlation > 0.7 between the centrality indices of the original sample and those of subset samples with 95% of possibility (37). In general, the CS-C value should be > 0.25, preferably > 0.5.

## Results

### Descriptive statistics

Table 1 presents the demographic characteristics of the study sample in this network analysis (*n* = 1830). The mean age was 20.1 years (SD = 1.2), 56.6% of the sample were from urban areas. The sample consisted of 30.5% males, 45.4% of them were sophomores, and approximately four-fifths (79.1%) had monthly disposable funds between 1001 and 2000 Yuan. The mean of BCEs total scores was 18.7 (SD = 1.8) and mean of uncertainty stress total scores was 23.2 (SD = 9.0). The specific information of the BCEs scale items and uncertainty stress scale items are shown in Supplementary Table 1.

**Table 1:** Demographic characteristics of the study sample (*n* = 1830).

**Variables**	**N**	**%**
**Residence status**		
Urban	1035	56.6
Rural	795	43.4
**Sex**		
Male	559	30.5
Female	1271	69.5
**Grade**		
Freshman	279	15.2
Sophomore	831	45.4
Junior	668	36.5
Senior	52	2.8
**Monthly disposable funds (RMB)**		
≤ 1000	93	5.1
1001–2000	1448	79.1
2001–3000	227	12.4
>3000	62	3.4
**Only child**		
yes	914	49.9
no	916	50.1
	**Mean**	**SD**
**Age (years)**	20.1	1.2
**Uncertainty stress total**	23.2	9.0
**BCEs total**	18.7	1.8

### Network structure and centrality measure analysis

**Figure 1:**
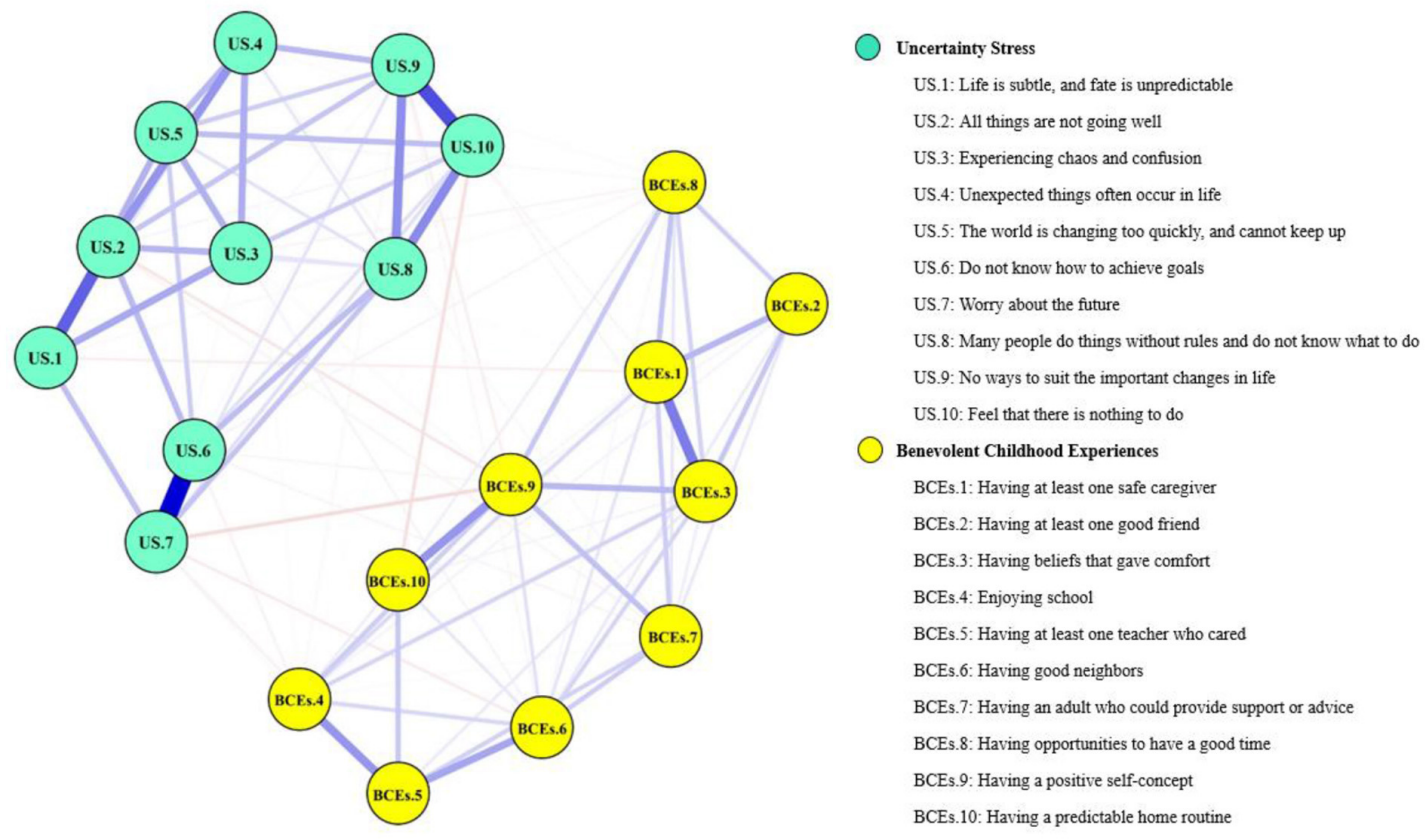
Network structure of BCEs and uncertainty stress among Chinese university students. The yellow nodes represent the BCEs items while the green nodes denote the uncertainty stress items. The closer the nodes are to each other, the stronger the connection. Meanwhile, blue edges represent positive correlations while red edges represent negative correlations. The magnitude of the correlation between nodes is represented by the edge thickness.

The network structure of BCEs and uncertainty stress among Chinese university students was shown in Figure 1 and the corresponding correlation matric is presented in Supplementary Table 2. Among the network of uncertainty stress (the left part of Figure 1), with all connections indicating positive associations. Of these, the thickest edge between the node US.6 (do not know how to achieve goals) and US.7 (worry about the future) means that the node US.6 (do not know how to achieve goals)-US.7 (worry about the future) has the strongest connection, followed by the node US.9 (no ways to suit the important changes in life)-US.10 (feel that there is nothing to do), and the node US.1 (life is subtle, and fate is unpredictable)-US.2 (all things are not going well). Regarding the network of BCEs (the right part of Figure 1), all the connections also showed positive associations between nodes. In the diagram, the edge between BCEs.1 (having at least one safe caregiver) and BCEs.3 (having beliefs that gave comfort) is the thickest, which means that BCEs.1 (having at least one safe caregiver)-BCEs.3 (having beliefs that gave comfort) has the strongest association. Besides, the node BCEs.9 (having at least one safe caregiver)-BCEs.10 (having beliefs that gave comfort), the node BCEs.4 (enjoying school) and the node BCEs.6 (having good neighbors) all have positive correlation with the node BCEs.5 (having at least one teacher who cared). Within the BCEs-uncertainty stress network model, the edge between the node US.7 (worry about the future) and BCEs.9 (having a positive self-concept) is the thickest, indicating that the node US.7 (worry about the future)-BCEs.9 (having a positive self-concept) has the strongest negative connection. Nodes US.10 (feel that there is nothing to do) and BCEs.10 (having a predictable home routine) were highly negative interconnected. Moreover, nodes US.7 (worry about the future) and BCEs.6 (having good neighbors), nodes US.1 (life is subtle, and fate is unpredictable) and BCEs.1 (having at least one safe caregiver) were also strongly negative interconnected.

**Figure 2:**
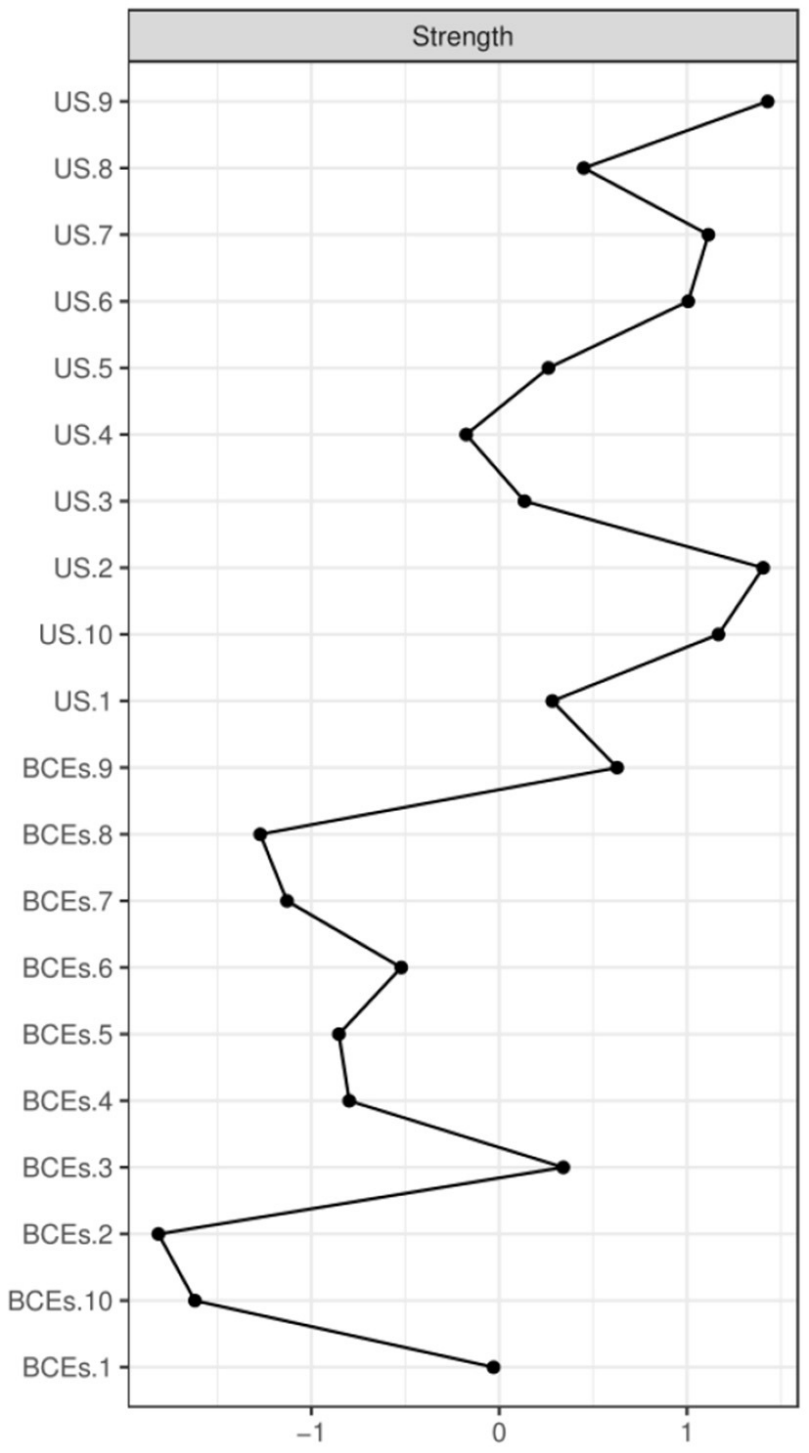
Centrality index of BCEs and uncertainty stress within the network, shown as standardized values z scores.

**Figure 3:**
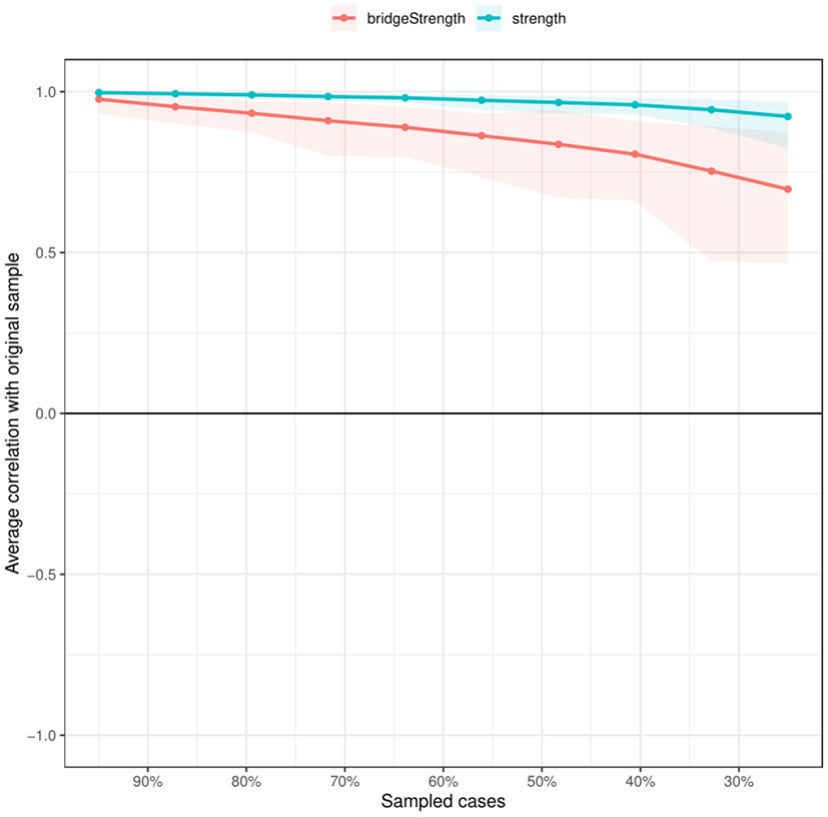
The case dropping subset bootstrap improves network structure stability. The x-axis shows the proportion of original sample cases included at each step. The y-axis shows the average of the correlations between the original network's centrality indices and the networks that were re-estimated after eliminating increasing percentages of cases.

In terms of strength, the node US.9 (no ways to suit the important changes in life) has the highest node strength in the BCEs-uncertainty stress network among Chinese university students, indicating that the node US.9 (no ways to suit the important changes in life) was the most influential, followed by nodes US.2 (all things are not going well), US.10 (feel that there is nothing to do), and US.7 (worry about the future) (Figure 2). On the contrary, several other symptoms were marginal, such as BCEs.8 (having opportunities to have a good time), BCEs.10 (having a predictable home routine) and BCEs.2 (having at least one good friend). In terms of network stability (Figure 3), the CS-coefficient of strength calculated by the case dropping bootstrap process was 0.75, indicating that the network remained stable, as eliminating 75% of the sample would not impact the primary results (r = 0.7).

### Network accuracy and stability

**Figure 4:**
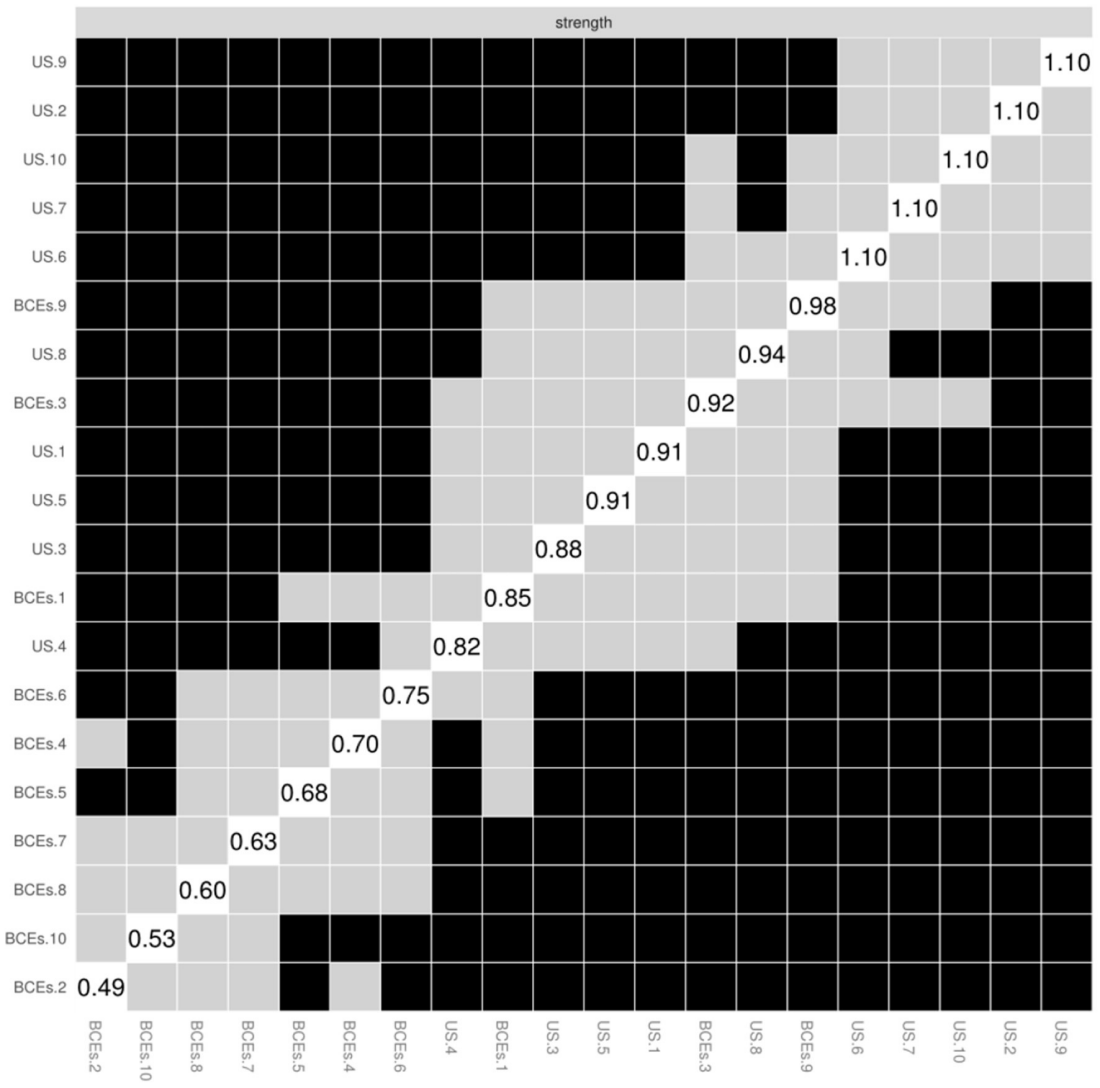
The node strength was tested using a non-parametric bootstrap difference test. Bootstrap difference tests between node strength of factors. Gray boxes denote nodes that do not significantly differ from one-another. Black boxes represent nodes that differ significantly from one another (α = 0.05). White boxes show the values of node strength.

Concerning the accuracy of the current network, the results of bootstrap 95% CI for edges demonstrate that 95% CIs were narrow, indicating that edges are trustworthy, as shown in Supplementary Figure 1. The network model was dependable and stable, according to the bootstrap 95% CIs for predicted edge-weights (Supplementary Figure 2). The bootstrap difference test revealed that the majority of the comparisons between edge weights were statistically significant (Figure 4).

### Bridge symptoms of BCEs and uncertainty stress

**Figure 5:**
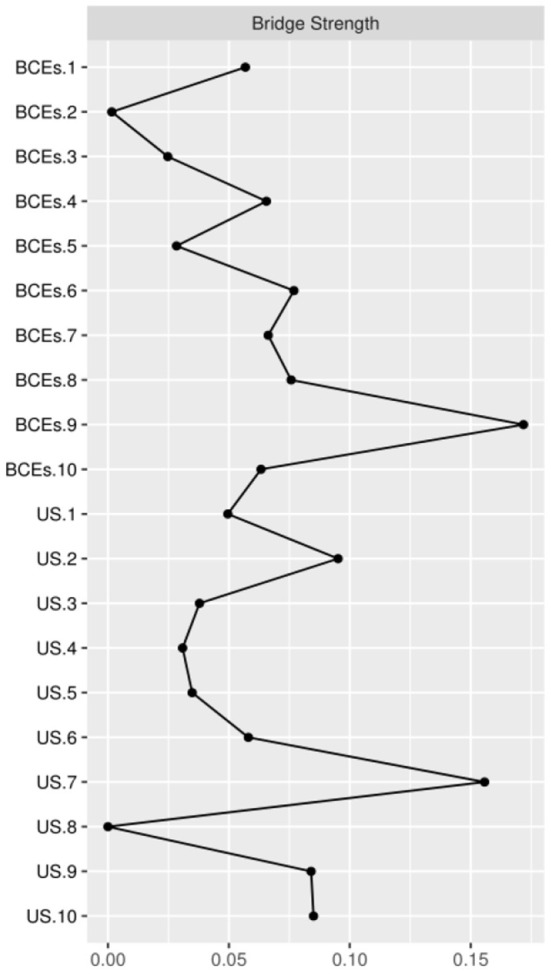
Bridge centrality indices of the BCEs-uncertainty stress network among Chinese university students.

The best metric for detecting nodes is bridge strength. In terms of bridge symptoms (Figure 5), BCEs.9 (having a positive self-concept) showed the highest bridge strength, followed by US.7 (worry about the future) and US.2 (all things are not going well).

## Discussion

### Summary of the results

To the best of our knowledge, this is the first exploratory study to use network analysis as a method of examining association between BCEs and uncertainty stress among Chinese university students. Analyses indicated the node US.9 (no ways to suit the important changes in life) was the most influential symptom within the BCEs- uncertainty stress network. Experiences and events disturb a person's established and routinized patterns of behavior, resulting in life changes (38). Uncertainty emerges in life when our sense of self-continuity is challenged by life changes. Changes lead us to re-evaluate who we are and who we will become as consequences of the changes in question (38). On the one hand, according to various researches, university students are presented with a variety of changes, including role shifts, interpersonal connections, and so on (39, 40). Adjustment disorders may contribute to freshmen students' uncertainty stress, especially when they were away from their parents and familiar friends and must adapt themselves to new situations (41). If uncertainty stress is not alleviated quickly, this emotional suppression can easily progress to mental diseases. On the other hand, the subjective well-being related to learning also represents a kind of life change (40). A study conducted in the UK indicated that students' well-being was put under a lot of stress after they started university (42). Moreover, given the structure of Chinese universities' curricula, which typically set general courses in the first year and progress to more specialized courses in the second year, students may face greater study pressure than in prior semesters. This may inadvertently contribute to an increase in uncertainty stress.

In this BCEs-uncertainty stress network, the most influential bridge symptom was “having a positive self-concept” (BCEs.9), where “having a positive self-concept” (BCEs.9) showed a high bridge centrality. When the genuine self is lost and an identity crisis arises, psychological difficulties occur, implying that self-concept should be a target of therapies to alleviate psychological disorders (43). It is critical to develop and maintain a positive self-concept, especially throughout adolescence, because it is related to a person's general health (44). Face-to-face interactions are difficult for some persons with low self-concept, causing them more difficult to socialize openly and directly, which could lead to development of uncertainty stress (45). Likewise, Xu et al. stated that the positive self-concept may help Chinese university students resist and effectively cope with the risk factors related to mental health (44). In other words, developing a positive self-concept is helpful for a happy life. Noteworthy, the strongest edge in the whole BCEs and uncertainty stress network was the connection between the “worry about the future” (US.7) and “having a positive self-concept” (BCEs.9), which could be due to the following reasons. Self-concept status is an uncertainty-relevant construct (46). Carleton et al. proposed that those who are lower in self-concept positive are more likely to worry about the future relative to individuals who are higher in self-concept positive (47). In addition, another study conducted in undergraduate students suggested that a moderate association between lower in self-concept positive and higher worry (48). Therefore, having a positive self-concept is an important role in one's own development for university students.

Not only for the university level, BCEs can have lifelong effects on mental health and physical health (27). BCEs improve individual's capacity to control emotions, which benefits adult health (49, 50). This skill, which is essential for daily functioning and has been linked to an exceptional skill to down-regulate stress and danger responses (51, 52). For example, positive childhood experiences such as social involvement and academic performance predicted much higher adult productivity and responsibility (53). In addition, Narayan et al. (24). demonstrated that greater levels of PCEs predicted fewer stressful life events during pregnancy and reduced posttraumatic stress disorder symptoms in a sample of low-income pregnant women. Moreover, the literature suggests that cardiovascular health scores were greater in those with higher scores on the indicator of happy childhood experiences (22). It is foreseeable that more cohort studies will be conducted in the future to explore how BCEs affect people's lives at different stages of life.

### Limitations of this study

Several limitations should be noted. First, due to the non-experimental, cross-sectional study design, causal relations between symptoms of BCEs and uncertainty stress could not be determined. Second, data were collected based on participants' self-report, and the possibility of recall bias could not be excluded. Third, the sample were only collected from three cities of China, thus the results may not representative of all university students in China. Although these limitations are existed, there is a big strength of this study should be noted. Unlike previous studies that used total scale scores to assess psychological status, the analytical approach in this study emphasized the association between each item within the scale and identified the most influential item between the scales, which is not widely used. Future research could begin with this approach, examining which items of the scale should be given more weight and determining which clinical interventions should be implemented first.

## Conclusions

The central symptoms of the BCEs-uncertainty stress network should be prioritized as targets in interventions and prevention efforts to reduce uncertainty stress among Chinese university students. Improving university students' positive self-concept is important to alleviate the level of uncertainty stress among Chinese university students.

## Data availability statement

The raw data supporting the conclusions of this article will be made available by the authors, without undue reservation.

## Ethics statement

The studies involving human participants were reviewed and approved by the Medical Ethics Committee of Xuzhou Medical University. Written informed consent to participate in this study was provided by the participants' legal guardian/next of kin.

## Author contributions

WW and CZ conceived the idea for the study. WW, YX, QZ, and CZ obtained the data. YP, WW, and JW cleared up the datasets. YP, WW, JT, NY, YL, and JW performed the data analyses. WW and YP interpreted the results of the data analyses and wrote the manuscript with the participation of other authors. All authors contributed to the article and approved the submitted version.

## Funding

This work was supported by the National Natural Science Foundation of China [82003484], Natural Science Fund for Colleges and Universities in Jiangsu Province [20KJB330005].

## Conflict of interest

The authors declare that the research was conducted in the absence of any commercial or financial relationships that could be construed as a potential conflict of interest.

## Publisher's note

All claims expressed in this article are solely those of the authors and do not necessarily represent those of their affiliated organizations, or those of the publisher, the editors and the reviewers. Any product that may be evaluated in this article, or claim that may be made by its manufacturer, is not guaranteed or endorsed by the publisher.

## Abbreviations

BCEs: between benevolent childhood experiences
eLASSO: Enhanced Least Absolute Shrinkage and Selection Operator
EBIC: Extended Bayes Information Criterion
CIs: confidence intervals
CS-C: Correlation Stability Coefficient.
