# Supplementary material for: Network connectivity between benevolent childhood experiences and uncertainty stress among Chinese university students
Source: Front Psychiatry. 2022 Nov 1;13:1007369. doi: 10.3389/fpsyt.2022.1007369 (PMC9665163; doi:10.3389/fpsyt.2022.1007369)
Supplement: Supplementary file 1 [file Data_Sheet_1.DOCX]

**Supplementary Table 1.** Mean, standard deviation, minima, maxima, skewness and kurtosis of BCEs and uncertainty stress (n=1830).

| **Items** | **M** | **SD** | **Min** | **Max** | **Skewness** | **Kurtosis** |
| --- | --- | --- | --- | --- | --- | --- |
| **Uncertainty stress** |  |  |  |  |  |  |
| 1.Life is subtle, and fate is unpredictable | 2.4 | 1.1 | 1 | 5 | 0.4 | -0.6 |
| 2.All things are not going well | 2.3 | 1.0 | 1 | 5 | 0.4 | -0.4 |
| 3.Experiencing chaos and confusion | 2.1 | 1.1 | 1 | 5 | 0.8 | -0.1 |
| 4.Unexpected things often occur in life | 2.2 | 1.0 | 1 | 5 | 0.6 | -0.1 |
| 5.The world is changing too quickly, and cannot keep up | 2.1 | 1.0 | 1 | 5 | 0.6 | -0.1 |
| 6.Do not know how to achieve goals | 2.6 | 1.2 | 1 | 5 | 0.2 | -0.7 |
| 7.Worry about the future | 2.7 | 1.2 | 1 | 5 | 0.2 | -0.7 |
| 8.Many people do things without rules and do not know what to do | 2.4 | 1.1 | 1 | 5 | 0.5 | -0.4 |
| 9.No ways to suit the important changes in life | 2.2 | 1.1 | 1 | 5 | 0.6 | -0.2 |
| 10.Feel that there is nothing to do | 2.2 | 1.1 | 1 | 5 | 0.7 | -0.1 |
| **BCEs** |  |  |  |  |  |  |
| 1.Having at least one safe caregiver | 1.9 | 0.2 | 1 | 2 | -3.8 | 12.5 |
| 2.Having at least one good friend | 1.9 | 0.1 | 1 | 2 | -6.6 | 42.1 |
| 3.Having beliefs that gave comfort | 1.9 | 0.2 | 1 | 2 | -3.6 | 10.9 |
| 4.Enjoying school | 1.8 | 0.4 | 1 | 2 | -1.6 | 0.4 |
| 5.Having at least one teacher who cared | 1.7 | 0.4 | 1 | 2 | -1.0 | -1.0 |
| 6.Having good neighbors | 1.8 | 0.4 | 1 | 2 | -1.8 | 1.1 |
| 7.Having an adult who could provide support or advice | 1.8 | 0.3 | 1 | 2 | -2.2 | 3.0 |
| 8.Having opportunities to have a good time | 1.9 | 0.2 | 1 | 2 | -5.5 | 28.4 |
| 9.Having a positive self-concept | 1.7 | 0.4 | 1 | 2 | -1.4 | 0 |
| 10.Having a predictable home routine | 1.8 | 0.3 | 1 | 2 | -1.6 | 0.7 |

**Note:** M: mean, SD: standard deviation, Min: minima, Max: maxima

**Supplementary Table 2.** Weighted adjacency matrix.

|  | BCEs.1 | BCEs.2 | BCEs.3 | BCEs.4 | BCEs.5 | BCEs.6 | BCEs.7 | BCEs.8 | BCEs.9 | BCEs.10 | US.1 | US.2 | US.3 | US.4 | US.5 | US.6 | US.7 | US.8 | US.9 | US.10 |
| --- | --- | --- | --- | --- | --- | --- | --- | --- | --- | --- | --- | --- | --- | --- | --- | --- | --- | --- | --- | --- |
| BCEs.1 | 0 | 0.149 | 0.274 | 0.076 | 0 | 0.053 | 0.100 | 0.121 | 0.017 | 0 | -0.034 | 0 | -0.005 | 0 | 0 | 0 | 0 | 0 | 0.018 | 0 |
| BCEs.2 | 0.149 | 0 | 0.115 | 0.022 | 0 | 0.042 | 0.047 | 0.101 | 0.017 | 0 | 0.002 | 0 | 0 | 0 | 0 | 0 | 0 | 0 | 0 | 0 |
| BCEs.3 | 0.274 | 0.115 | 0 | 0.087 | 0.042 | 0.085 | 0.034 | 0.091 | 0.148 | 0.021 | 0 | 0 | 0 | 0 | 0 | 0 | -0.005 | 0 | -0.002 | -0.018 |
| BCEs.4 | 0.076 | 0.022 | 0.087 | 0 | 0.219 | 0.083 | 0.009 | 0 | 0.102 | 0.034 | 0 | -0.026 | 0 | -0.008 | 0 | 0 | -0.012 | 0 | -0.016 | -0.005 |
| BCEs.5 | 0 | 0 | 0.042 | 0.219 | 0 | 0.184 | 0.092 | 0 | 0.002 | 0.116 | -0.001 | 0 | 0 | 0 | 0 | 0 | -0.027 | 0 | 0 | 0 |
| BCEs.6 | 0.053 | 0.042 | 0.085 | 0.083 | 0.184 | 0 | 0.103 | 0.015 | 0.060 | 0.048 | 0 | 0 | 0 | 0 | 0.020 | 0 | -0.039 | 0 | -0.017 | 0 |
| BCEs.7 | 0.100 | 0.047 | 0.034 | 0.009 | 0.092 | 0.103 | 0 | 0.051 | 0.127 | 0 | 0 | -0.020 | -0.012 | -0.014 | 0 | -0.020 | 0 | 0 | 0 | 0 |
| BCEs.8 | 0.121 | 0.101 | 0.091 | 0 | 0 | 0.015 | 0.051 | 0 | 0.115 | 0.031 | -0.013 | 0 | -0.021 | -0.010 | -0.015 | 0.018 | 0 | 0 | 0 | 0 |
| BCEs.9 | 0.017 | 0.017 | 0.148 | 0.102 | 0.002 | 0.060 | 0.127 | 0.115 | 0 | 0.219 | 0 | -0.049 | 0 | 0 | 0 | -0.018 | -0.072 | 0 | -0.032 | 0 |
| BCEs.10 | 0 | 0 | 0.021 | 0.034 | 0.116 | 0.048 | 0 | 0.031 | 0.219 | 0 | 0 | 0 | 0 | 0 | 0 | -0.001 | 0 | 0 | 0 | -0.062 |
| US.1 | -0.034 | 0.002 | 0 | 0 | -0.001 | 0 | 0 | -0.013 | 0 | 0 | 0 | 0.333 | 0.179 | 0.055 | 0.160 | 0 | 0.133 | 0 | 0 | 0 |
| US.2 | 0 | 0 | 0 | -0.026 | 0 | 0 | -0.020 | 0 | -0.049 | 0 | 0.333 | 0 | 0.153 | 0.220 | 0 | 0.145 | 0 | 0.048 | 0.121 | 0.017 |
| US.3 | -0.005 | 0 | 0 | 0 | 0 | 0 | -0.012 | -0.021 | 0 | 0 | 0.179 | 0.153 | 0 | 0.171 | 0.161 | 0 | 0.010 | 0.052 | 0.011 | 0.106 |
| US.4 | 0 | 0 | 0 | -0.008 | 0 | 0 | -0.014 | -0.010 | 0 | 0 | 0.055 | 0.220 | 0.171 | 0 | 0.150 | 0 | 0 | 0.046 | 0.146 | 0 |
| US.5 | 0 | 0 | 0 | 0 | 0 | 0.020 | 0 | -0.015 | 0 | 0 | 0.160 | 0 | 0.161 | 0.150 | 0 | 0.105 | 0 | 0.058 | 0.103 | 0.134 |
| US.6 | 0 | 0 | 0 | 0 | 0 | 0 | -0.020 | 0.018 | -0.018 | -0.001 | 0 | 0.145 | 0 | 0 | 0.105 | 0 | 0.537 | 0.138 | 0.010 | 0.062 |
| US.7 | 0 | 0 | -0.005 | -0.012 | -0.027 | -0.039 | 0 | 0 | -0.072 | 0 | 0.133 | 0 | 0.010 | 0 | 0 | 0.537 | 0 | 0.121 | 0.054 | 0.064 |
| US.8 | 0 | 0 | 0 | 0 | 0 | 0 | 0 | 0 | 0 | 0 | 0 | 0.048 | 0.052 | 0.046 | 0.058 | 0.138 | 0.121 | 0 | 0.236 | 0.244 |
| US.9 | 0.018 | 0 | -0.002 | -0.016 | 0 | -0.017 | 0 | 0 | -0.032 | 0 | 0 | 0.121 | 0.011 | 0.146 | 0.103 | 0.010 | 0.054 | 0.236 | 0 | 0.373 |
| US.10 | 0 | 0 | -0.018 | -0.005 | 0 | 0 | 0 | 0 | 0 | -0.062 | 0 | 0.017 | 0.106 | 0 | 0.134 | 0.062 | 0.064 | 0.244 | 0.373 | 0 |

**Note:** Weighted adjacency matrix based on factors to represent the weight of direct edges between nodes; US: uncertainty stress


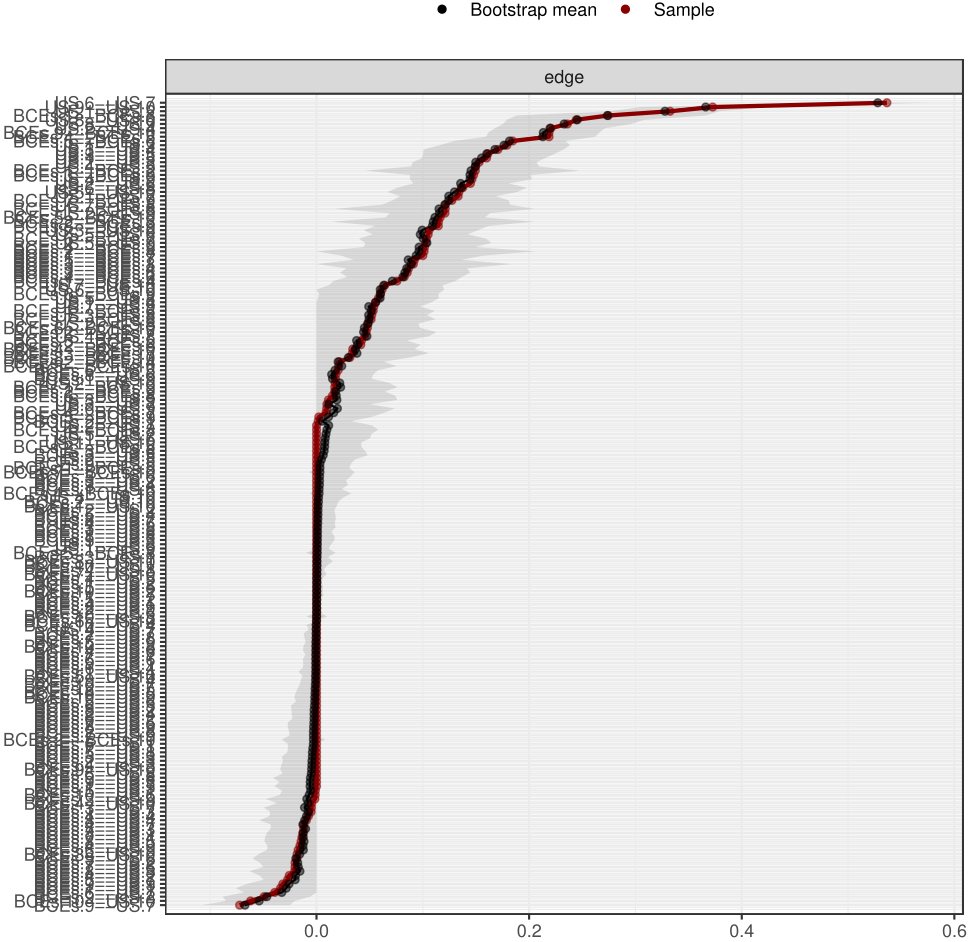


**Supplementary Figure 1.** Bootstrap confidence intervals of edge weights. The y-axis represents the edges of the network model, arranged from top to bottom in the same order as the original edges. The black dots indicate the values of each edge weight, ordered from the highest to the lowest value. The gray area represents the 95% Confidence Intervals of edge weights, estimated with the non-parametric bootstrap procedure (“Bootnet” package). Wide intervals indicate lower stability and narrow intervals indicate higher stability.


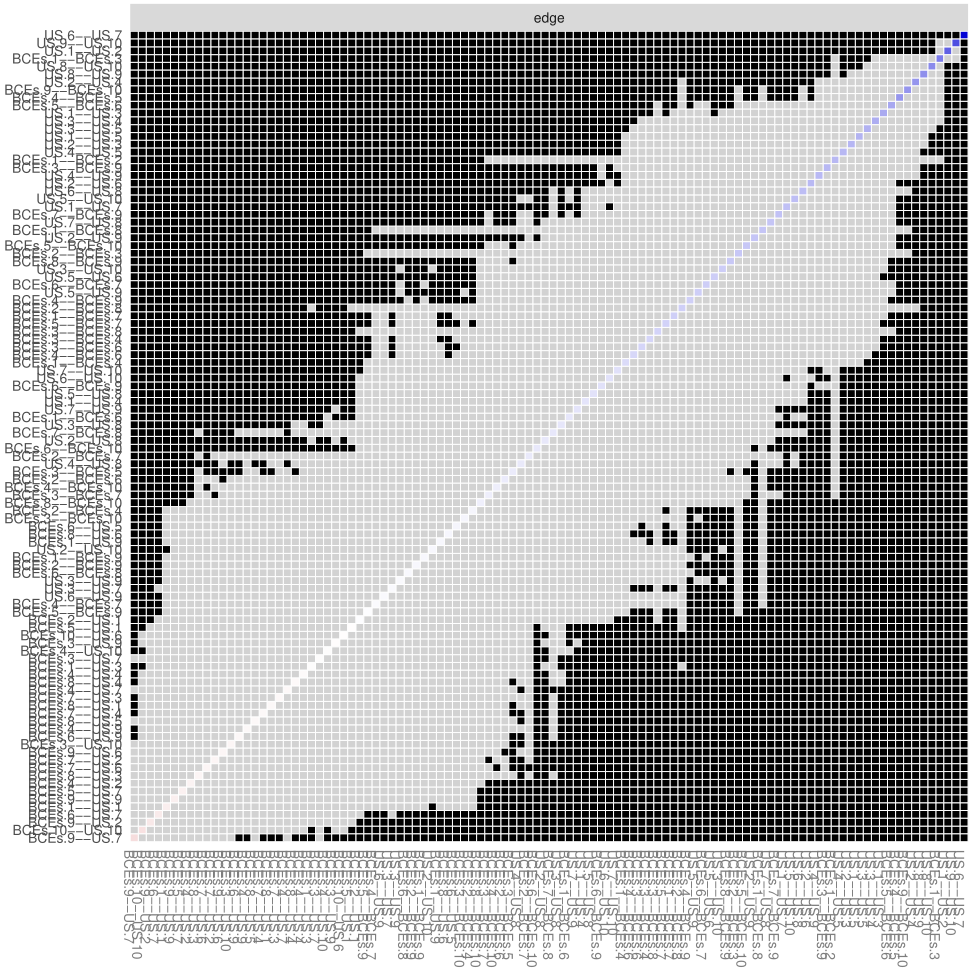


**Supplementary Figure 2.** Estimation of edge weight difference by bootstrap difference test. Bootstrap difference tests between edge weights in the network. Both x-axis and y-axis represent the edges of the network model. Gray boxes indicate edges that do not significantly differ from one-another. Black boxes represent edges with significant difference from one another (α=0.05). Blue boxes in the edge-weight plot indicate positive correlations.
